# Plasmid DNA-coding p62 as a bone effective anti-inflammatory/anabolic agent

**DOI:** 10.18632/oncotarget.2884

**Published:** 2015-02-12

**Authors:** Maria Giovanna Sabbieti, Dimitrios Agas, Melania Capitani, Luigi Marchetti, Antonio Concetti, Cecilia Vullo, Giuseppe Catone, Vladimir Gabai, Victor Shifrin, Michael Y Sherman, Alexander Shneider, Franco M Venanzi

**Affiliations:** ^1^ School of Biosciences and Veterinary Medicine, University of Camerino, Camerino (Italy); ^2^ CureLab Oncology Inc, 43 Rubury Hillway Needham MA (USA); ^3^ Dept. Biochem, Boston University School of Medicine, Boston MA (USA)

**Keywords:** p62/SQSTM1, chronic inflammation, osteoporosis, immunotherapy

## Abstract

We recently reported that a DNA plasmid coding p62-SQSTM1 acts as an effective anti tumor vaccine against both transplantable mouse tumors and canine spontaneous mammary neoplasms. Here we report the unexpected finding that intramuscular delivery of p62 DNA exerts a powerful anti-osteoporotic activity in a mouse model of inflammatory bone loss (i.e, ovariectomy) by combining bone-sparing and osteo-synthetic effects. Notably, the suppression of osteoporosis by p62DNA was associated with a sharp down-regulation of master inflammatory cytokines, and up-regulation of endogenous p62 protein by bone-marrow stromal cells. The present data provide a solid rational to apply p62 DNA vaccine as a safe, new therapeutic for treatment of inflammatory related bone loss diseases.

## INTRODUCTION

The adapter protein p62 (also known as sequestosome1/SQSTM1) is a multifunctional molecule involved in a myriad of cellular processes that modulate proliferation, cell death, inflammation and immune response [1]. Further studies demonstrated that p62 plays an important role in patho-physiology of human diseases including neurodegenerative diseases, lung disease, obesity, insulin resistance, cancer, and Paget's disease of bone [2]. Since p62 holds a key role in innate immunity by regulating inflammatory signaling cascades, the hypothesis that deregulated p62 is linked to the chronic allostasis that foster dreadful inflammatory disorders is gaining ground [3, 4]. Accordingly, p62 has been envisaged as a potential target in cancer, infectious and inflammatory diseases [5].

We first reported that p62 could be a target for cancer immunotherapy. Indeed, we provide evidence that DNA plasmid-encoded human p62 triggered effective anti-tumor/antimetastatic activities in four models of allogenic mouse tumors (i.e. melanoma, lung carcinoma, sarcoma, and breast cancer) [6]. We also demonstrated that p62 DNA plasmid when administered in neo-adjuvant (pre-operative) setting decreased and/or stabilized growth of advanced lesions in canine mammary tumors [7].

Although there are no *in vivo* data regarding the role of exogenous p62 in inflammation, there are some *in vitro* evidence suggesting that p62 may act as an important regulator of cytokine expression [8–11]. In example, in activated macrophages p62 overexpression and underexpression clearly lead to opposite effects pointing to the inhibitory role of p62 in cytokine expression, thus providing a mechanism by which p62 controls excessive inflammatory responses [11]. Of much interest, is a recent report that highlights the anti-inflammatory tumor suppressor potential of p62, as its down-regulation in the tumor stroma fosters an inflammatory response that enhances tumorigenesis both *in vitro* and *in vivo* [12].

The existence of a causal link between inflammation and p62 is further supported by the observation that the lack of a p62 (or macroautophagy) contributes to inflammation in aging [4]. The term “inflammaging” was coined at the beginning of this century by Franceschi et al. [13] to refer to a low-grade pro-inflammatory phenotypes which accompany aging in mammals and that predispose the organism to develop several age-related diseases.

Osteoporosis is, by far, the leading age-related disease affecting mostly women after onset of menopause. Because estrogens contribute to bone-sparing activity by inhibition of bone remodeling (coupled with a balancing effect on bone formation and resorption), decreased levels of circulating estrogen at menopause results in a rapid bone loss [14]. Further observations indicate that the estroprivic bone loss reflects increased number of T cells, that in concert with macrophages and bone marrow-derived stromal cells promote the release of pro inflammatory cytokines (e.g TNF-α and IL-1β beta, IL-6, and RANK-L) that propel osteoclastogenesis, and thereby bone erosion [15] (See Supplementary Figure 1). Finally, the strong link between inflammation and osteoporosis is further evidenced by clinical studies showing that chronic inflammatory diseases of almost any cause, are associated with bone loss (osteopenia) [16].

Based on the putative anti-inflammatory role of p62, together with the fact that p62 physiologically controls osteoclastogenesis and bone remodeling [17], we hypothesized that p62 DNA vaccine could compensate for osteoporosis. To test the hypothesis we carried out experiments by injecting p62 DNA in ovariectomized (OVX) mice. Since ovariectomy induces chronic inflammation in mice and in humans [18], this animal model is widely utilized to study premature aging [19], and to test experimental anti-osteoporotic drugs and biologicals [20].

The results shown below are representative of those obtained by in independent trials carried out both in preventive and therapeutic settings.

## RESULTS AND DISCUSSION

### p62 DNA administration prevents osteoporosis in OVX mice

**Figure 1:**
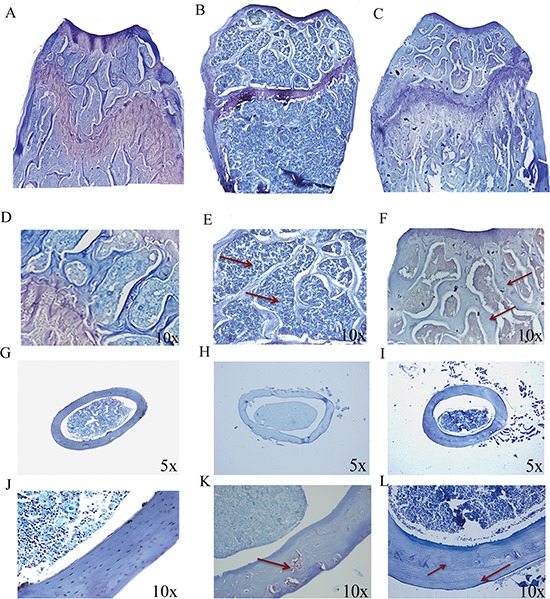
p62 DNA prevents osteoporosis Representative reconstructions of metaphyseal regions of the distal femurs from sham operated **(A, D)**, pcDNA3.1-OVX **(B, E)** and p62 DNA-OVX mice **(C, F)**. Arrows indicated the thinner and reduced trabecular network in pcDNA3.1-OVX mice **(E)** compared with that observed in p62 DNA-OVX mice **(F)** Representative section of femur mid diaphysis from sham operated **(G, J)**, pcDNA3.1-OVX **(H, K)** and p62 DNA-OVX mice **(I, L)**. Arrows indicated cortical bone porosity in pcDNA3.1-OVX mice **(K)** and the new bone apposition in p62 DNA-OVX mice **(L)** Magnifications: 10 × (D, E, F, J, K, L), 5 × (G, H. I).

To evaluate whether p62 vaccine was able to prevent osteoporosis, groups of mice were first injected either with experimental (p62DNA) or reference plasmids (pcDNA 3.1) and then ovariectomized (OVX). For each trial a control group of sham operated (SO) mice was included (see M&M for details). Two months after surgery mice were sacrificed, and the collected long bones subjected to histological examination. As expected, the metaphyseal regions of the distal femurs from pcDNA3.1-OVX mice displayed classical osteoporotic features characterized by significant bone loss and thinned disconnected trabecular structure (Figure 1B, 1E). On the other hand, p62-OVX bones (Figure 1C, 1F) showed a micro-architecture essentially indistinguishable to that seen in SO mice (Figure 1A, 1D). Moreover, examination of cross sections femur diaphysis from p62DNA-OVX mice revealed (at variance of those obtained from reference plasmids treated mice) an enhanced anabolic - osteoblastic activity as evidenced by new cortical bone apposition suggesting an anabolic action of p62 treatment. (Figure 1G–1L).

**Figure 2:**
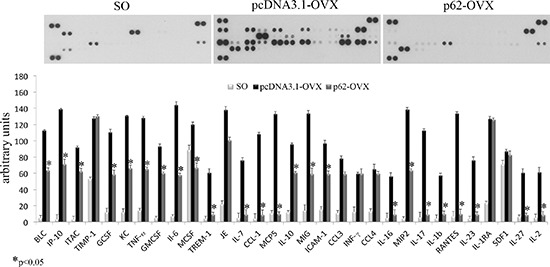
p62 DNA pretreatment decreases pro-inflammatory cytokines and chemokines release in OVX mice Cytokines and chemokines release was analyzed in medium from bone marrow cultures obtained by sham operated, p62 or pcDNA 3.1 pre-treated OVX mice. (*p < 0.05).

**Figure 3:**
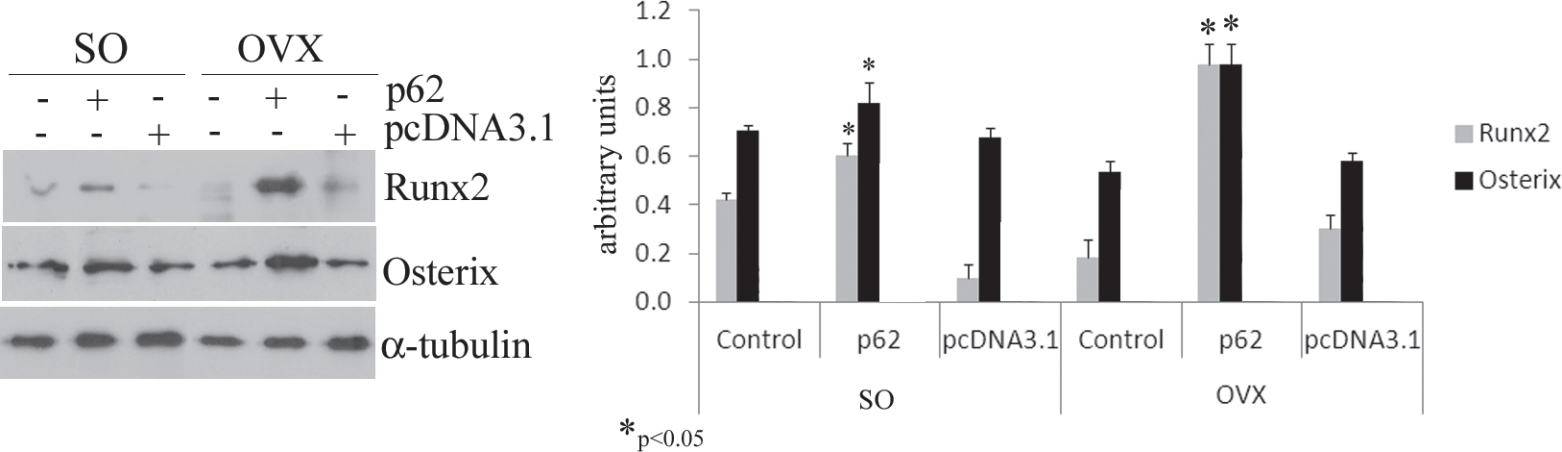
p62 DNA increases osteogenic markers Total bone marrow cell populations were obtained from long bones of sham operated, pcDNA3.1-OVX and p62 DNA-OVX mice and were examined by Western blotting for Runx2 and osterix expression. (*p < 0.05).

Fully consistent with the morphological findings are the biochemical results obtained by utilizing bone marrow stromal cells (BMCs) retrieved from plasmids pre-treated mice. In these experiments BMCs were flushed from the bone cavities, and cultured for 3 days. Afterwards, both supernatants and cells were collected and analyzed respectively either for the release of inflammatory cytokines, or for expression of osteogenic markers. As illustrated in Figure 2, we observed that the marked up-regulation and release of pro-inflammatory cytokines by BMCs from OVX compare to SO operated mice was drastically suppressed by p62-DNA pretreatment. The inhibitory effect of p62 DNA extended to an array of cytokines such as TNFα, IL-6, IL-1b IL-17, all known to be essential inducers of inflammatory diseases and bone loss [21]. As far as the capability of p62DNA to induce new bone formation is concerned, western blotting analysis of p62-OVX BMCs extracts indicated a strong and selective increase of osteogenic markers, such as Runx2 and Osterix transcription factors [22]. An increase of Runx2 and Osterix, although weaker, was also found in p62 SO mice (Figure 3).

### p62 DNA as an anti-osteoporosis therapeutic

**Figure 4:**
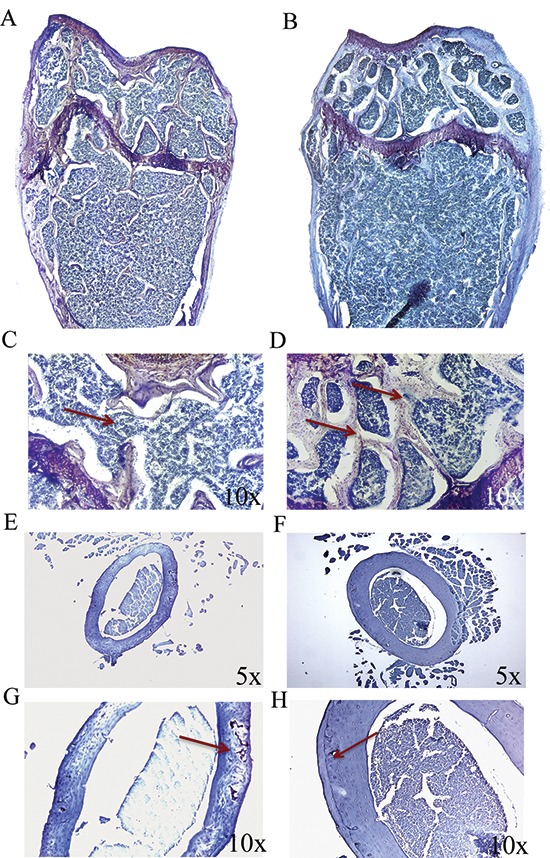
p62 DNA rescues osteoporosis Representative reconstructions of metaphyseal regions of distal femurs from OVX-pcDNA3.1 **(A, C)** and OVX-p62DNA mice **(B, D)**. Arrows indicated the trabecular bone loss **(C)** and the restored trabecular microarchitecture **(D)** Representative sections of femur mid diaphysis from OVX-pcDNA3.1 **(E, G)** and OVX-p62 DNA mice **(F, H)**. Note the expansion of the medullary cavity and the resorption cavities within the cortex (Arrow, G). Arrows indicated the reconstitute cortical bone structure **(H)** Magnifications: 10 × (C, D, G, H), 5 × (E, F).

**Figure 5:**
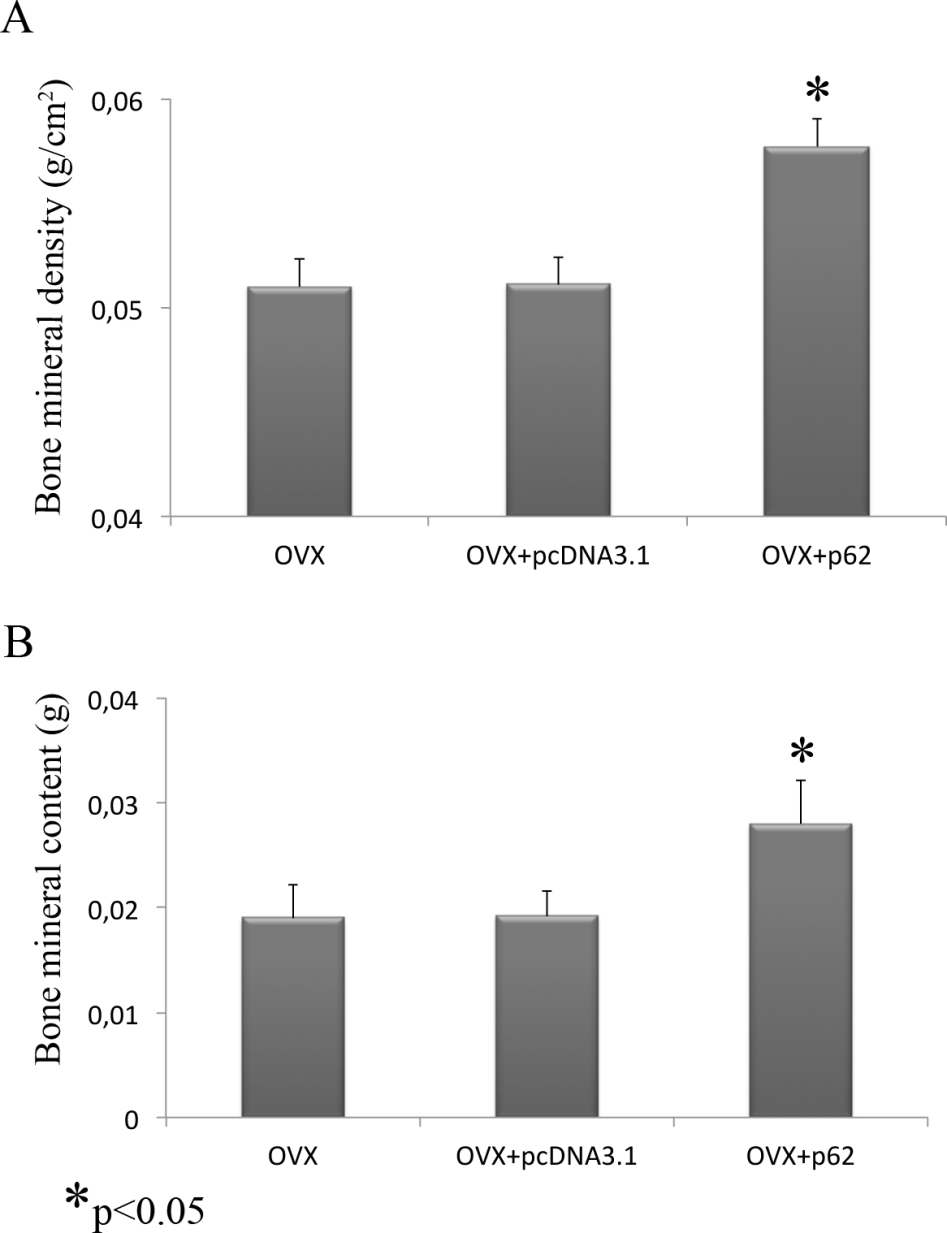
BMD and BMC evaluation after p62 treatment Bone mineral density **(A)** and bone mineral content **(B)** were increased in OVX mice treated with p62-encoding plasmid. (*p < 0.05).

**Figure 6:**
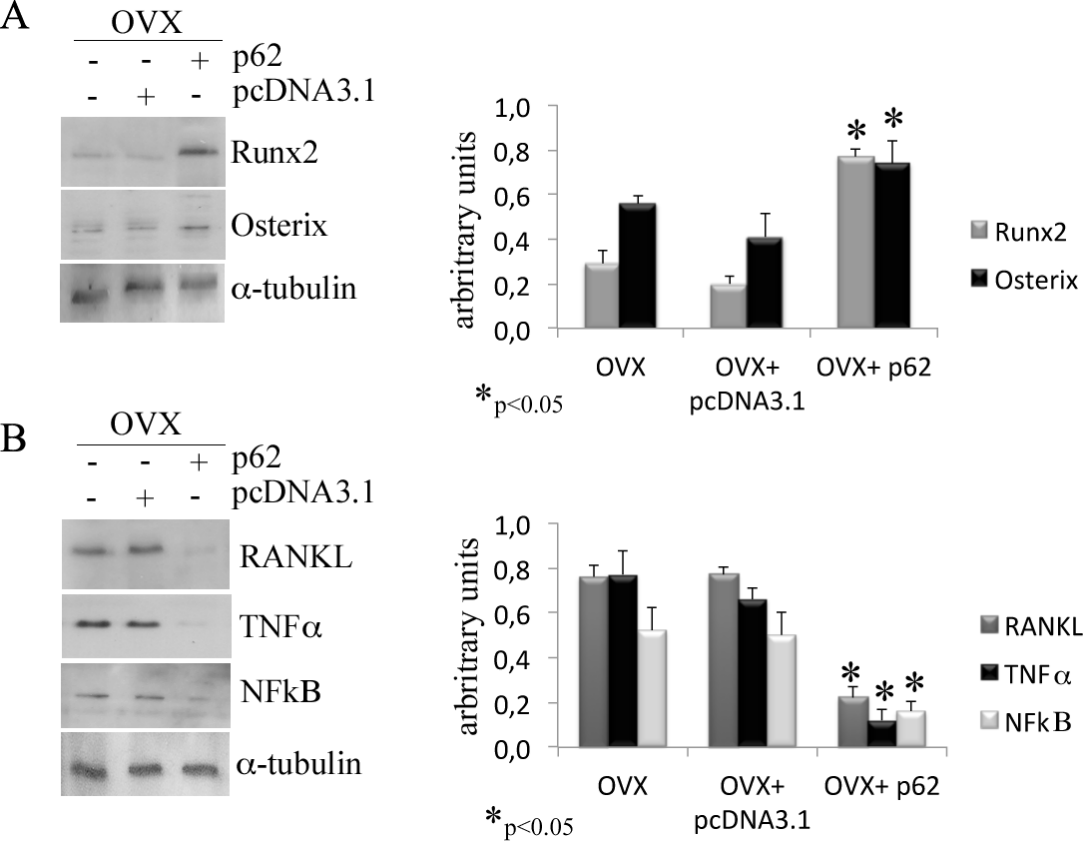
p62 vaccine decreases pro-inflammatory markers Total bone marrow cell population was obtained from long bones of OVX, OVX-pcDNA3.1 and OVX-p62 DNA. Note the statistically significant increase of Runx2 and osterix **(A)** as well as the decrease of RANKL, TNFα and NFkB **(B)** protein levels only after p62 treatment. (*p < 0.05).

After establishing that p62 DNA vaccine demonstrates robust effects not only in preventing osteoporosis but also in inducing new bone formation, we assessed the therapeutic potential of p62 DNA in case of established osteoporosis. In these trials, mice were ovariectomized and, after two months, injected either with p62-DNA or reference plasmids (see M&M for details). Two months after last plasmids injections, bones were collected and histologically evaluated. In this set of experiments we reproducibly found that OVX-p62 treated mice group (at variance of control groups) showed a restored trabecular microarchitecture at metaphyseal regions of the distal femurs and a decreased porosity in cortical bone (Figure 4A–4H). In addition, p62-DNA treatment proved to increase both bone mineral density (BMD) and content (BMC) as judged by DEXA analysis (Figure 5A–5B). Finally, coupled with marked up-regulation of osteoblastogenic Runx2 and Osterix (Figure 6A), a strong inhibition of two majors bone resorptive factors such as TNFα and RANKL was also observed in BMCs from OVX-p62 mice. It is worth noting that RANKL is a key mediator of inflammation that, by binding to its receptor RANK on osteoclast precursors, fosters osteoclastogenesis via intracellular NF-kB signaling [23]. In our experimental setting, a down-regulation of NF-kB in OVX-p62 BMCs was also observed (Figure 6B).

### A unexpected ring: exo-p62 up-regulates endo-p62 expression

**Figure 7:**
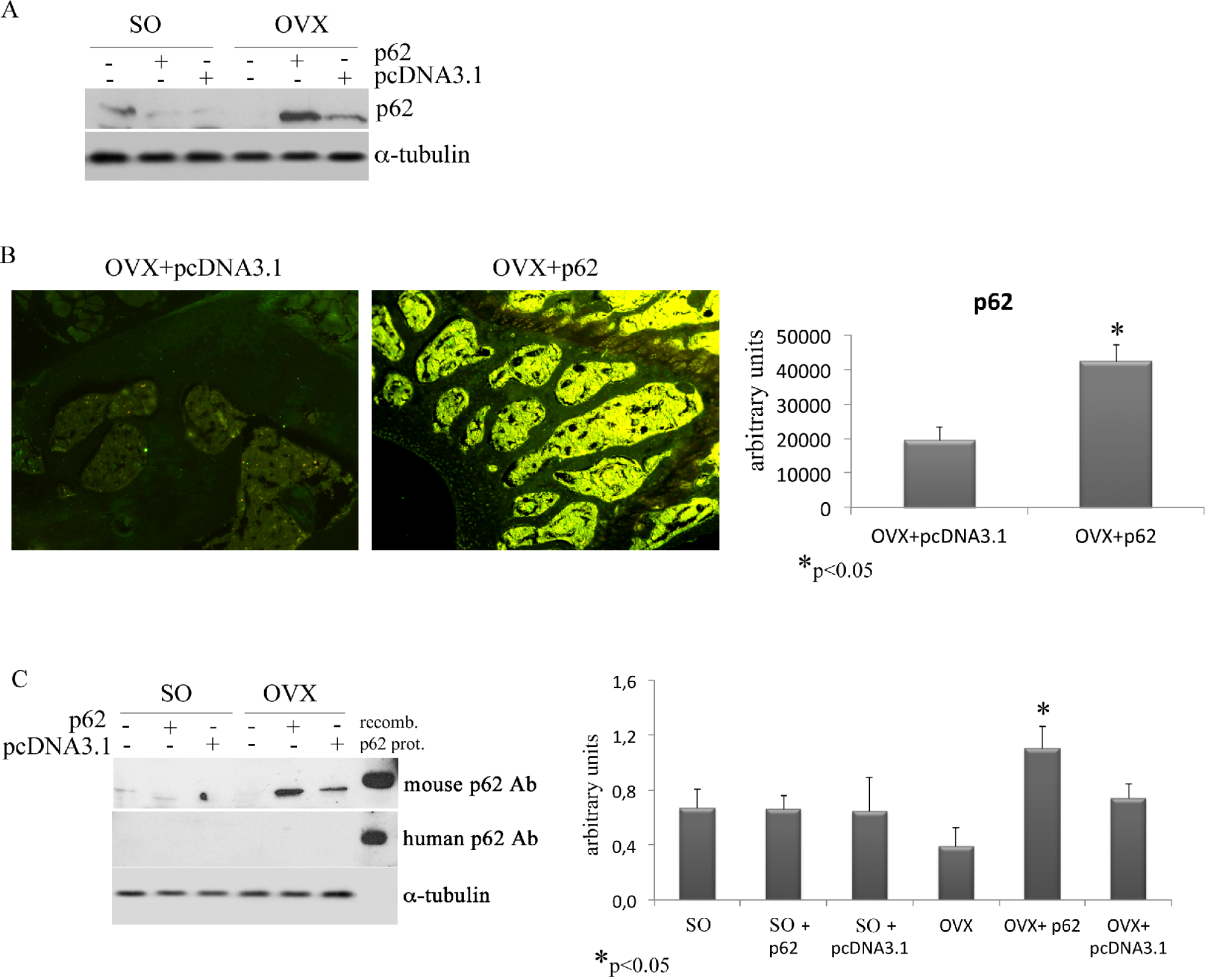
p62 encoding plasmid up-regulates the endogenous p62 protein synthesis p62 protein levels were detected in total bone marrow population **(A)** and in the epiphyseal areas of femur sections **(B)** by Western blotting and immunofluorescence, respectively. Note the increase of p62 synthesis in samples from p62-OVX mice. Evaluation of p62 levels in BMCs by a specific mouse/human antibody or by a specific human antibody. Note the lack of human p62 detection, and the increase of mouse p62 protein in BMCs from p62-OVX mice **(C)** (*p < 0.05).

As anticipated, although there are reports in the literature suggesting that *in vitro* overexpression of p62 may dull the production of inflammatory cytokines [11], there are no *in vivo* data about a putative role of exogenous p62 DNA in inflammation. While not wishing to be held by theory, we evaluated the expression levels of p62 in BMCs retrieved from plasmids injected in mice before ovariectomy. With much of our surprise, we found that while p62 expression in BMCs was down-regulated by ovariectomy, BMCs from p62 DNA pre-injected mice demonstrated a sturdy and selective up-regulation of p62 (Figure 7A). Consistently, an increased p62-immune labeling was observed at the epiphyseal region of femurs of p62-OVX mice (Figure 7B).

Next we asked whether this enhanced p62 immune-reactivity refers to exogenous human p62DNA or to endogenous mouse p62. In order to distinguish between these two possibilities we utilized two types of anti-p62 antibodies for western blotting analysis of BMCs extracts: a polyclonal rabbit Ab that recognized both human and mouse p62, and a monoclonal Ab that recognize only human p62 [24]. A human p62 recombinant protein was loaded as positive control. As depicted in (Figure 7C), we found that p62DNA administration clearly up-regulates endogenous p62 protein in bone marrow resident cells.

A growing understanding of the bone remodeling processes suggest that factor involved in inflammation are linked with those critical for bone physiology and remodeling, supporting the theory that inflammation significantly contributes to the aetiopathogenesis of osteoporosis. Indeed, our results provide for the first time evidences that in this model (OVX) of inflammation-mediated osteopenia, p62DNA, by exerting powerful anti-inflammatory effects, counteracts bone loss, and as consequence, promotes new bone formation [25]. Importantly, the dramatic effects of p62 DNA on bone homeostasis were not associated with any side effects as demonstrated by previous long-term toxicological studies [26]. In this context it has to be underlined that the major pharmacological interventions for osteoporosis, so far proposed, evidenced a long list of worrisome side effects [27, 28].

Taken together, our data can be viewed as proof of concept for pre-clinical and clinical development of an unprecedented strategy based on p62-DNA to counteract osteoporosis [29]. More broadly, we hypothesize that the administration of p62 DNA may quench chronic inflammatory reactions underpinning a variety of age-related diseases.

## MATERIALS & METHODS

### DNA Plasmids

Human p62 (SQSTM, isoform 1) was cloned in pcDNA3.1 (InVitrogene) vector as previously described [6]. Large scale preparations of the endotoxin-free plasmids were routinely performed by alkaline lysis using either Endo Free Plasmid Kit (Qiagen) or Gen Elute HPSelect Plasmid Giga Prep columns (SIGMA # NA0800). For intramuscular injections (femoral quadriceps), mice were anesthetized and injected with 100 μg DNA (1 mg/ml) in saline solutions. All groups were subjected to three injections at one week intervals.

### Animals and treatments

Thee-month old female FVB and Balb/c mice (Harlan Italy SrL, Correzzana Milano, Italy) were used. Mice were kept in laminar-flow cage in a standardized environmental condition. In prevention trials mice were randomly distributed in three groups (G1–G3) and injected intramuscularly at week 0, 1, 2 with only saline (G1, *n* = 12), with pcDNA3.1 (G2, *n* = 12), or with hp62 DNA (G3 *n* = 12). At day forty-five after the last injection, mice from each group were randomly divided in two subgroups and were sham operated (SO; *n* = 6) or ovariectomized (OVX *n* = 6). After two months mice were sacrificed by CO2 narcosis according to the recommendation of the Italian Ethical Committee. For therapeutic trials mice were ovariectomized (OVX) and left untreated for 2 months. Afterwards, mice were randomized in 3 groups, and injected with plasmids as described above. After 2 months mice were sacrificed for analysis.

### Histological bone analysis and immuflourescence

Femurs, dissected of adhering tissue, were fixed in 4% paraformaldehyde (PFA) for 24 h, decalcified in 14% EDTA solution for 3 days and soaked in 30% sucrose overnight. Samples, embedded with Tissue-Tek OCT compound, were sectioned (8 μm thick sections) by a rotatory −30°C microtome cryostat (Ames Cryostat Miles) and stained with toluidine blue.

Other sections, after permeabilization with 0.3% Triton X-100 were incubated with rabbit anti-p62 diluted 1: 800 (Enzo Life Sciences; Vinci-Biochem s.r.l., Firenze, Italy) diluted 1:400. After rinsing, sections were incubated with chicken anti-rabbit IgG Alexa Fluor 488 conjugated (Molecular Probes; Invitrogen, Milano Italy) diluted 1:100. Control experiments were performed by omitting the appropriate primary antibody or by neutralizing the primary antibodies with the relative blocking peptide. Slides were imaged using a Leica DM 2500 fluorescent microscopy. Fluorescence analysis was performed by a fluorimeter Tecan Infinite [30].

### *Ex vivo* dual-energy X-ray absorptiometry (DEXA) analyses

Femurs were dissected and fixed as above described. Bone mineral density (BMD) and bone mineral content (BMC) were measured using a PIXImus DEXA [31].

### Bone marrow cell (BMCs) preparation

Long bones (femurs, tibiae and humeri) from the above mouse groups were dissected free of adhering tissue. The ends were removed and the marrow cavity was flushed and cultured in DMEM as previously described [32].

### Cytokines and chemokines assay

The cytokine/chemokine profiles of BMCs supernatants were assessed by ELISA-based cytokine array by using Mouse Cytokine Array Panel A kit (R&D Systems, Milano, Italy) accordingly to the manufacturer's instructions. Immunoreactive dots were visualized using LiteAblot Turbo luminol reagents (Euroclone, Milano, Italy) and Hyperfilm-ECL film (Euroclone, Milano, Italy) and quantitated densitometrically.

### Western blotting

Proteins from total bone marrow cells population were extracted in Cell Lysis Buffer (Cell Signaling Euroclone, Milano, Italy) immediately after flushing the bone marrow cavity, and the concentration was determined by the BCA protein assay reagent (Pierce, Euroclone Milano, Italy). Western blotting was performed as previously described [33]. Human recombinant p62 was purchased from Enzo Life Sciences; Vinci-Biochem s.r.l., Firenze, Italy. Membranes were immunoblotted in blocking buffer with specific antibodies: rabbit anti-mouse p62 (1: 800 dilution, Enzo Life Sciences; Vinci-Biochem s.r.l., Firenze, Italy); mouse anti-human p62 (1:800 dilution, BD Transduction Laboratories, Milano, Italy); mouse anti-Receptor activator of NF-κB ligand (RANKL) antibody (1:250 dilution, Abcam, Prodotti Gianni, Milano, Italy); rabbit anti-Runx-2 antibody (1:800 dilution, Cell Signaling, Euroclone, Milano, Italy); rabbit anti-Osterix antibody (1:600 dilution, Santa Cruz Biotechnology, Italy); rabbit anti-NF-kB (1:500 dilution, BioLegend, Microtech Srl, Napoli, Italy). After washing blots were incubated with horseradish peroxidase (HRP)-conjugated donkey anti-rabbit IgG or with HRP-conjugated rabbit anti-mouse IgG (Cell Signaling, Euroclone Milano, Italy). Immunoreactive bands were visualized using luminol reagents/ECL film as described above. To normalize the bands, filters were stripped and re probed with a monoclonal anti-α-tubulin (Sigma-Aldrich, Milano, Italy). Bands density was quantified densitometrically.

### Statistical analysis

All *in vitro* and *in vivo* experiments were repeated at least three times. t-student was used to test for significant differences between two groups, and differences were considered significant at (*p < 0.05).

## SUPPLEMENTARY FIGURE


