# Supplementary material for: Plasmid DNA-coding p62 as a bone effective anti-inflammatory/anabolic agent
Source: Oncotarget. 2015 Feb 12;6(6):3590–9. doi: 10.18632/oncotarget.2884 (PMC4414139; doi:10.18632/oncotarget.2884)
Supplement: Supplementary file 1 [file oncotarget-06-3590-s001.pdf]

SUPPLEMENTARY FIGURE

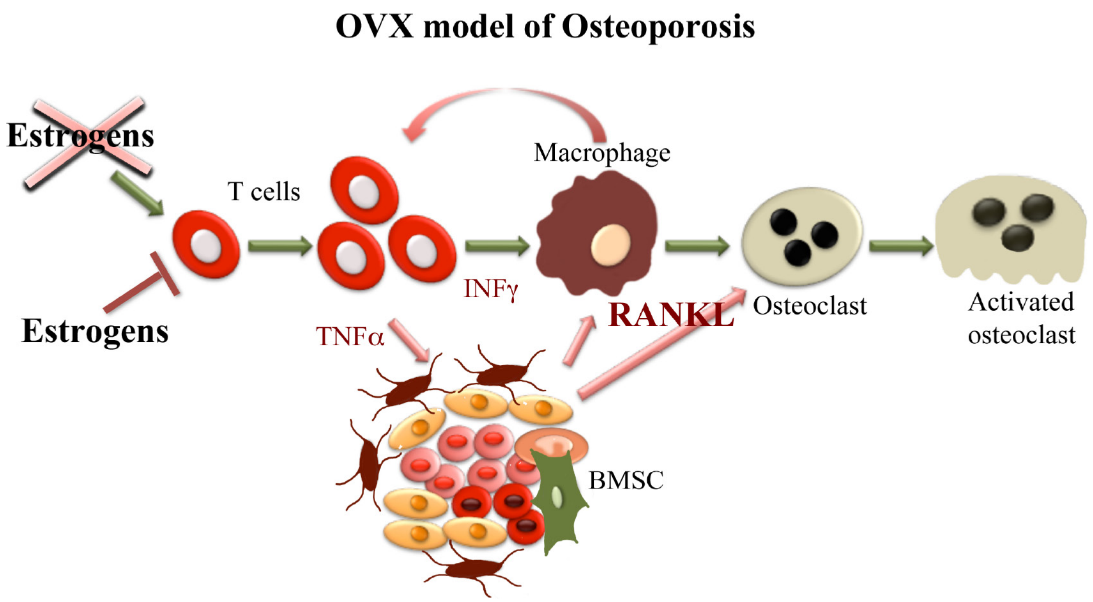

Supplementary Figure 1: Graphic illustration of the role of T cells in the mechanism by which ovx supports osteoclastogenesis. After ovariectomy estrogen deficiency promotes T cell activation within the bone marrow. Activated T cells secrete TNFα, which coupled with RANKL secretion stimulates osteoclast formation and concurrently increase M-CSF, and other pro-inflammatory factors [16].
